# Altered NKp30, NKp46, NKG2D, and DNAM-1 Expression on Circulating NK Cells Is Associated with Tumor Progression in Human Gastric Cancer

**DOI:** 10.1155/2018/6248590

**Published:** 2018-09-03

**Authors:** Bin Han, Fang-yuan Mao, Yong-liang Zhao, Yi-pin Lv, Yong-sheng Teng, Mubing Duan, Weisan Chen, Ping Cheng, Ting-ting Wang, Zhong-yuan Liang, Jin-yu Zhang, Yu-gang Liu, Gang Guo, Quan-ming Zou, Yuan Zhuang, Liu-sheng Peng

**Affiliations:** ^1^Department of Pharmacy, Affiliated Hospital of North Sichuan Medical College, Nanchong, Sichuan Province 637000, China; ^2^National Engineering Research Center of Immunological Products, Department of Microbiology and Biochemical Pharmacy, College of Pharmacy, Third Military Medical University, Chongqing 400038, China; ^3^Department of General Surgery and Center of Minimal Invasive Gastrointestinal Surgery, Southwest Hospital, Third Military Medical University, Chongqing 400038, China; ^4^La Trobe Institute for Molecular Science, La Trobe University, Bundoora, VIC 3085, Australia

## Abstract

Natural killer (NK) cell activity is influenced by a complex integration of signaling pathways activated downstream of both activating and inhibitory surface receptors. The tumor microenvironment can suppress NK cell activity, and there is a great clinical interest in understanding whether modulating tumor-mediated NK cell suppression and/or boosting preexisting NK cell numbers in cancer patients is therapeutically viable. To this light, we characterized the surface receptor phenotypes of peripheral blood NK cells and examined their clinical relevance to human gastric cancer (GC). We found that the proportion of peripheral blood NK cells which expressed the activating receptors NKp30, NKp46, NKG2D, and DNAM-1 was significantly decreased in GC patients compared to healthy donors, and that this decrease was positively associated with tumor progression. At the same time, plasma TGF-*β*1 concentrations were significantly increased in GC patients and negatively correlated with the proportion of NKp30, NKp46, NKG2D, and DNAM-1 expressing NK cells. Furthermore, TGF-*β*1 significantly downregulated the expression of NKp30, NKp46, NKG2D, and DNAM-1 on NK cells *in vitro*, and the addition of galunisertib, an inhibitor of the TGF-*β* receptor subunit I, reversed this downregulation. Altogether, our data suggest that the decreased expression of activating receptors NKp30, NKp46, NKG2D, and DNAM-1 on peripheral blood NK cells is positively associated with GC progression, and that TGF-*β*1-mediated NK cell suppression may be a therapeutically targetable characteristic of GC.

## 1. Introduction

Natural killer (NK) cells are important components of innate immunity and constitute the first line of host defense against tumors. Increased NK-mediated immune response has been positively associated with cancer patient outcomes [1, 2]. However, accumulating evidence also indicates that tumor-infiltrating NK cells can be functionally altered by the tumor microenvironment and conversely contribute to tumor growth and progression [3, 4]. Therefore, in-depth characterization of NK cell phenotypes and functionality may help address whether simply boosting NK cell expansion or simultaneously removing prior immunosuppressive factors will benefit the development of effective NK-targeted immunotherapies against cancer.

NK cell activation and lysis of target cells are determined by both the loss of inhibitory receptor-mediated signaling and presence of activating receptor-mediated signaling. The balance between these two signaling pathways determines the functional profile of NK cells [5, 6]. NK cell function is triggered by the loss inhibitory receptor-mediated signaling when tumor cells downregulate the expression of MHC Class I. These NK cell inhibitory receptors are the killer-cell immunoglobulin-like receptors (KIRs) in humans, and include CD158a/h, CD158b, CD158e1, and the killer cell lectin-like receptor subfamily C, member 1 (CD94/NKG2A) [7]. The engagement of activating receptors such as NKG2D, DNAM-1, and the natural cytotoxicity receptors NKp30, NKp44, and NKp46 is still required for the robust activation of NK cells and NK cell-mediated killing of transformed cancer cells [8, 9]. Thus, the characterization of NK cell surface receptors can be a helpful indicator of their functional, including antitumoral, capacity.

Gastric cancer (GC) is one of the most common malignancies worldwide with poor prognosis [10]. The clinical outcome of GC can be influenced by crosstalk between the tumor and host immune system [11] with our previous study demonstrating that the presence of tumor-infiltrating NK cells with impaired effector functions contributed to a decrease in GC survival [3]. Recently, NK cells in the peripheral blood of human cancer patients were reported to downregulate activating receptors NKG2D, NKp30, and DNAM-1 expression, and low levels of these receptors were positively associated with disease progression [12]. In GC, decreased expression of NKG2D and NKp30 in peripheral blood NK cells was also observed in patients with advanced disease [13], suggesting that tumor progression is associated with the loss of NK cell activating receptor expression. However, the mechanisms driving the systemic regulation of NK cell phenotypes, including activating receptor expression levels, remain elusive for GC.

In the present study, we characterized the surface receptor phenotypes of circulating NK cells in human GC and found that the proportion of peripheral blood NK cells which expressed the activating receptors NKp30, NKp46, NKG2D, and DNAM-1 was significantly decreased in GC patients compared to healthy donors, and that this effect could be mediated *in vitro* by TGF-*β*1. Therefore, targeting TGF-*β*1 signaling may be an important therapeutic strategy for reversing tumor-mediated immune escape of NK cells during GC.

## 2. Materials and Methods

### 2.1. Patients and Samples

Fresh peripheral blood was collected from 30 patients with GC undergoing surgery at the Southwest Hospital of the Third Military Medical University. None of the patients had received chemotherapy or radiotherapy during sampling. The clinical stages of tumors were determined according to the TNM classification system of the International Union Against Cancer (Edition 7). Blood from 30 age- and sex-matched healthy donors was collected as the control group. The study was approved by the Ethics Committee of the Third Military Medical University. Written informed consent was also obtained from each subject. The clinical characteristics of patients with GC were presented in Supplementary Table 1.

### 2.2. Cell Cultures

Peripheral blood mononuclear cells (PBMCs) were isolated by Ficoll-Paque density gradient centrifugation, and NK cells were then purified from the PBMCs of healthy donors by negative selection using the EasySep human NK-cell enrichment kit (Stem cell, Vancouver, Canada). Purified NK cells were resuspended at 2 × 10^5^ cells/well in RPMI 1640 containing 10% fetal calf serum (FCS) and seeded in 96-well plates supplemented with 1000 U/ml recombination human (rh) IL-2 (rhIL-2) to each well in the presence or absence of 10 ng/ml rhTGF-*β*1 (Peprotech, Rocky Hill, NJ). In some cases, NK cells were treated with 5 *μ*M of the TGF-*β* receptor I inhibitor galunisertib (MedChem Express, Monmouth Junction, NJ) for 1 hour followed by stimulation with 10 ng/ml rhTGF-*β*1. After 48 hours, plated cells were harvested and stained with antibodies (listed below) for flow cytometry analysis.

### 2.3. Antibodies and Flow Cytometry

The following antibodies were used to stain single cell suspensions: CD3-APC (UCHT1), CD56-PE-Cy7 (MEM-188), DNAM-1-FITC (TX25), CD16-FITC (3G8), CD94-FITC (DX22), CD158a/h-FITC (HP-MA4), CD158e1-FITC (DX9), NKG2D-PE (1D11), NKp30-PE (P30-15), 2B4-PE (C1.7), CD158b-PE (DX27), NKp46-PercP-Cy5.5 (9E2) (BioLegend, San Diego, CA), and NKG2A-PE (131411) (R&D system, Minneapolis, MN). Briefly, plated cells or RBC lysed peripheral whole blood cells were incubated with antibodies for 30 min at 4°C, then washed with PBS, and fixed for 20 min using 4% paraformaldehyde. Cells were further washed twice with PBS and analyzed by flow cytometry using a FACS Canto II (BD Biosciences). Isotype antibodies were used as controls. Data was analyzed using the Flowjo software (BD Biosciences).

### 2.4. Enzyme-Linked Immunosorbent Assay (ELISA)

Plasma from GC patients was collected, and plasma TGF-*β*1 concentrations were determined using ELISA kits (Dakewe Bioengineering Co., LTD, Shenzhen, China) according to the manufacturer's instructions.

### 2.5. Statistical Analysis

Results were summarized as mean ± standard error of the mean (SEM), and statistical analysis was performed with the Prism 5.0 software. The correlation analysis between different groups was determined by the Spearman's correlation test. Student's *t*-test was used to evaluate the differences between two groups. *P* < 0.05 was considered as statistically significant.

## 3. Results

### 3.1. GC Patients Exhibit a Decreased Proportion of NKp30, NKp46, NKG2D, and DNAM-1 Expressing Peripheral Blood NK Cells

**Figure 1:**
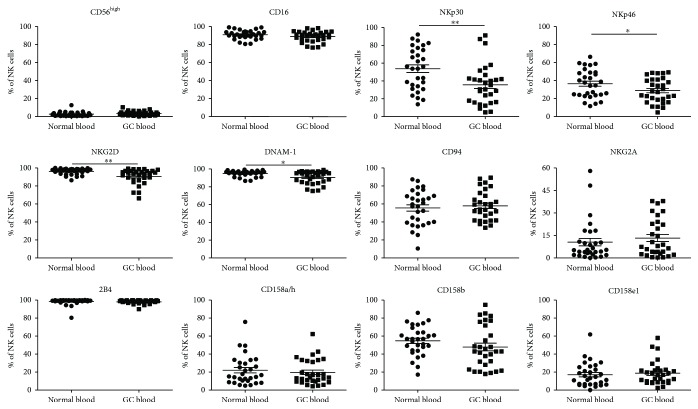
Phenotypic analysis of circulating NK cells in GC patients. Human peripheral whole blood from GC patients were stained with anti-CD3, anti-CD56, anti-CD16, anti-NKp30, anti-NKp46, anti-NKG2D, anti-DNAM-1, anti-2B4, anti-CD94, anti-NKG2A, anti-CD158a/h, anti-CD158b, and anti-CD158e1 antibodies or isotype controls. CD3^−^CD56^+^ NK-cell subpopulation was gated, and then, the levels of CD56^high^, CD16^+^, NKp30^+^, NKp46^+^, NKG2D^+^, DNAM-1^+^, CD94^+^, 2B4^+^, NKG2A^+^, CD158a/h^+^, CD158b^+^, and CD158e1^+^ cells in NK cells were analyzed. Data were expressed as the mean ± SEM. ^∗^ *P* < 0.05; ^∗∗^ *P* < 0.01.

We first characterized the proportion of NK cells from the peripheral blood of GC patients. CD3^−^CD56^+^ NK cells, CD3^+^CD56^+^ NKT cells, and CD3^+^CD56^−^ T cells were analyzed from the lymphocyte gate as defined by FSC and SSC properties (Supplementary Figure 1). No significant differences in the percentages of these cell subsets were observed between GC patients and healthy donors. However, in comparison to healthy donors, the percentages of CD3^−^CD56^+^ NK cells which expressed the activating receptors NKp30, NKp46, DNAM-1, and NKG2D were significantly decreased in GC patients (Figure 1). The expression of other peripheral blood NK cell surface receptors including CD16, CD94, NKG2A, 2B4, CD158a/h, CD158b, and CD158e1 was not significantly altered between GC patients and healthy donors (Figure 1 and Table 1). Thus, our results indicated that the proportion of peripheral blood NK cells which expressed the activating receptors NKp30, NKp46, DNAM-1, and NKG2D was decreased in GC patients.

We then determined the function profile of NK cells from the peripheral blood of GC patients. NK cells from GC patients exhibited a lower degranulation potential (CD107a expression) against K562 cells than those in healthy donors (Supplementary Figure 2). In addition, the percentage of perforin expression NK cells in GC patients was also significantly decreased, and its level positively correlated with the proportion of NKp30, NKp46, NKG2D, and DNAM-1 expressing NK cells (Supplementary Figure 3), suggesting that NK cell functional impairment is linked to their decreased expression of activating receptors.

### 3.2. Increased Plasma TGF-*β*1 Levels Negatively Correlate with the Proportion of NKp30, NKp46, NKG2D, and DNAM-1 Expressing Peripheral Blood NK Cells in GC Patients

**Figure 2:**
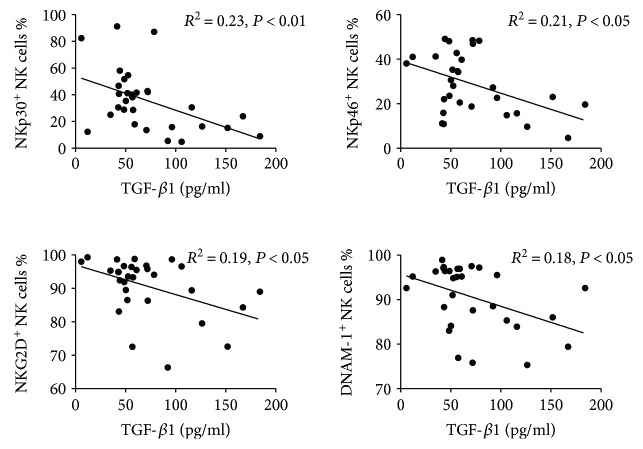
Correlation of TGF-*β*1 levels with the percentages of NKp30^+^, NKp46^+^, NKG2D^+^, and DNAM-1^+^ NK cells in GC patients. The concentrations of plasma TGF-*β*1 in GC patients were negatively correlated with the percentages of NKp30^+^, NKp46^+^, NKG2D^+^, and DNAM-1^+^ NK cells. *P* < 0.05 was considered to be significant of correlation between the two groups.

TGF-*β*1 is a key immunomodulatory cytokine which can regulate NK cell surface receptor expression and function. Given that decreased proportions of activating receptor expressing NK cells were observed in the peripheral blood of GC patients, we measured the plasma concentration of TGF-*β*1 in GC patients. Expectedly, the concentration of plasma TGF-*β*1 was significantly higher in GC patients compared to healthy donors (Supplementary Figure 4). Next, we studied the relationships between plasma TGF-*β*1 concentration and activating receptors expressing peripheral blood NK cell levels in these patients. Plasma TGF-*β*1 levels negatively correlated with the proportion of NKp30 and NKp46 expressing peripheral blood NK cells, and an inverse linear association was also observed between plasma TGF-*β*1 levels and the proportion of NKG2D and DNAM-1 expressing peripheral blood NK cells (Figure 2). These data suggest that the systemic downregulation of activating receptors NKp30, NKp46, NKG2D, and DNAM-1 expression NK cell levels may be linked to an aberrant increase in plasma TGF-*β*1 levels during GC.

### 3.3. TGF-*β*1 Downregulates NKp30, NKp46, NKG2D, and DNAM-1 Expression Levels on NK Cells *In Vitro*

**Figure 3:**
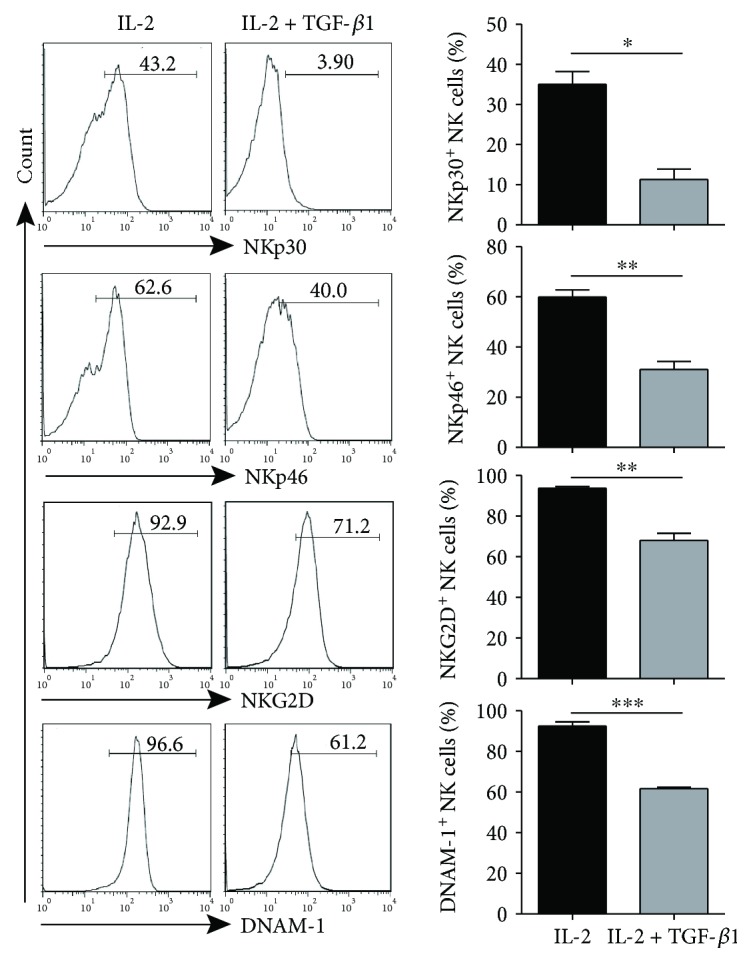
TGF-*β*1-dependent downregulation of NKp30, NKp46, NKG2D, and DNAM-1 expression on NK cells. Purified NK cells from healthy donors were seeded in 96-well plates supplemented with 1000 U/ml rhIL-2 for 48 hours with or without 10 ng/ml rhTGF-*β*, and the expression of NKp30, NKp46, NKG2D, and DNAM-1 on NK cells were detected by flow cytometry (*n* = 5). Left panel: a representative analysis, right panel: statistical analysis. ^∗^ *P* < 0.05; ^∗∗^ *P* < 0.01; ^∗∗∗^ *P* < 0.001.

**Figure 4:**
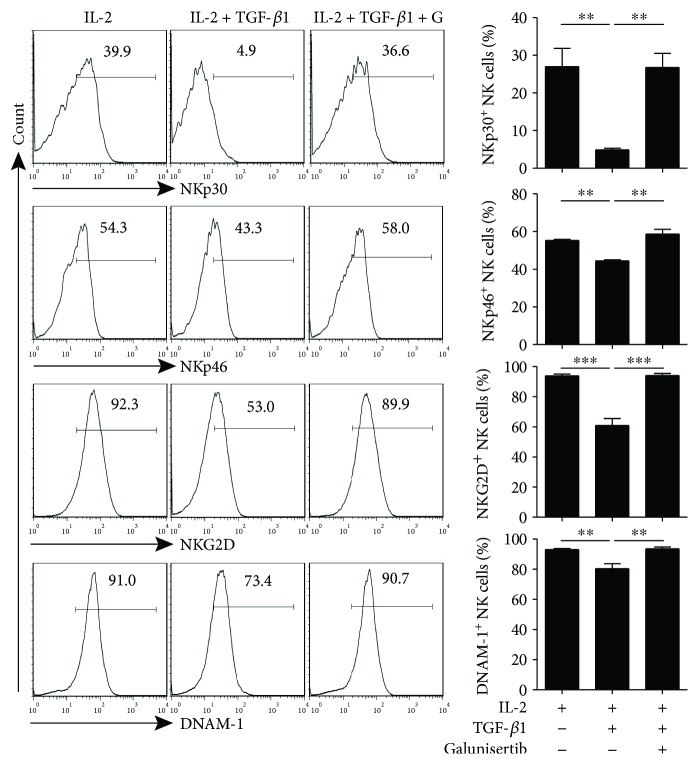
Galunisertib reversed TGF-*β*1-dependent downregulation of NKp30, NKp46, NKG2D, and DNAM-1 expression on NK cells. Purified NK cells from healthy donors were treated with 5 *μ*M TGF-*β* receptor I inhibitor galunisertib (G) for 1 hour followed by 10 ng/ml rhTGF-*β*1 stimulation. After 48 hours, the expression of NKp30, NKp46, NKG2D, and DNAM-1 on NK cells were detected by flow cytometry (*n* = 5). ^∗∗^ *P* < 0.01; ^∗∗∗^ *P* < 0.001.

To directly study the effects of TGF-*β*1 on the cell surface expression of activating receptors on NK cells, freshly purified NK cells from the peripheral blood of healthy donors were stimulated with or without TGF-*β*1. After 48 hours of culture, TGF-*β*1 significantly downregulated the expression of NKp30, NKp46, NKG2D, and DNAM-1 on NK cells (Figure 3). The expression of other NK cell surface receptors such as CD16, CD94, NKG2A, 2B4, CD158a/h, CD158b, and CD158e1 was not altered (Supplementary Figure 5). These data indicated that TGF-*β*1 could selectively downregulate NKp30, NKp46, NKG2D, and DNAM-1 expression on NK cells without altering the expression of other NK cell receptors. Next, we investigated whether galunisertib (a TGF-*β*R1 inhibitor) could abrogate the suppressive effect of TGF-*β*1. The downregulation of NKp30, NKp46, NKG2D, and DNAM-1 expression on NK cells by TGF-*β*1 was reversed when cells were pretreated with galunisertib, further indicating that the targeting of TGF-*β*1 could alter NK cell activating receptor NKp30, NKp46, NKG2D, and DNAM-1 expression levels (Figure 4).

### 3.4. Decreased Proportions of NKp30, NKp46, NKG2D, and DNAM-1 Expressing Peripheral Blood NK Cells and Increased Plasma TGF-*β*1 Levels Positively Correlate with GC Tumor Stage

**Figure 5:**
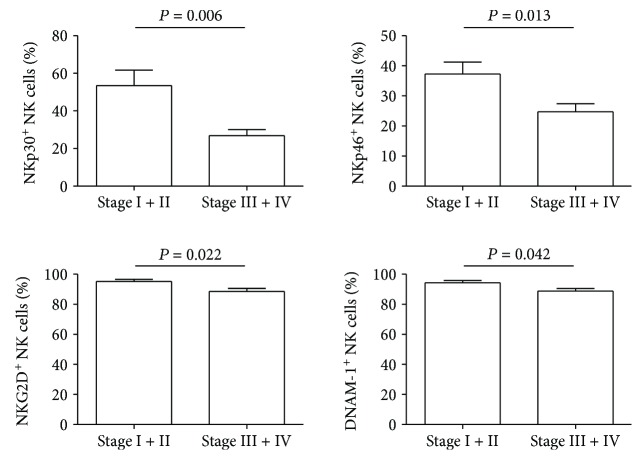
Correlation of the percentages of NKp30^+^, NKp46^+^, NKG2D^+^, and DNAM-1^+^ NK cells with GC stages. The percentages of NKp30^+^, NKp46^+^, NKG2D^+^, and DNAM-1^+^ NK cells between stage I-II and stage III-IV of GC patients were compared. *P* < 0.05 was considered to be significant.

Finally, we studied whether there were any relationships between the proportions of NKp30, NKp46, NKG2D, and DNAM-1 expressing peripheral blood NK cells, plasma TGF-*β*1 levels, and tumor progression. The comparison was made between stage I-II and stage III-IV GC patients. There was a significant difference in the proportions of NKp30, NKp46, NKG2D, and DNAM-1 expressing peripheral blood NK cells between stage I-II and stage III-IV GC patients, and NK cell receptor expression levels were negatively associated with tumor stage (Figure 5). At the same time, increased plasma TGF-*β*1 levels were shown to be positively associated with tumor stage (Supplementary Figure 6). Altogether, these results suggest that increased plasma TGF-*β*1 levels and an associated decrease in the expression levels of NKp30, NKp46, NKG2D, and DNAM-1 on NK cells contribute to GC progression.

## 4. Discussion

NK cell function is governed by a complex integration of both activating and inhibitory receptor-mediated signaling pathways. Optimal function requires both the absence of inhibitory receptor-mediated signaling and the engagement of activating receptor-mediated signaling, respectively [14]. In the present study, we showed that the proportion of peripheral blood NK cells which expressed the activating receptors NKp30, NKp46, NKG2D, and DNAM-1 was significantly decreased in GC patients compared to healthy donors and also negatively correlated with GC progression. Additionally, the addition of TGF-*β*1 was sufficient to downregulate NK cell activating receptors expression levels *in vitro.* This reflects an important mechanism whereby downregulation of NK cell activating receptor levels, and hence NK cell function, may contribute to tumor immune escape in GC.

In humans, the characterization of immune cell phenotypes that indicates functional immunological status has been greatly beneficial for the understanding of immune cell heterogeneity in cancer patients [15–17]. Our previous studies have showed that NK cell and NKT cell infiltration was significantly decreased in the GC microenvironment, and that their levels were strongly associated with disease progression and patients' survival [3, 18]. However, the proportion of these cell subsets in the peripheral blood of GC patients was comparable to healthy donors, suggesting that no alterations in the proportion of circulating NK and NKT cells are observed during GC progression. Upon further characterization, however, we observed a significant decreased in the proportion of peripheral blood NK cells which expressed the activating receptors NKp30, NKp46, NKG2D, and DNAM-1 in GC patients. Given that the dynamic expression of both activating and inhibitory receptors is crucial for optimal NK cell-mediated tumor cytotoxicity, decreased activating receptor expression levels are likely to contribute to impaired NK cell functionality [19, 20]. Actually, peripheral blood NK cells in GC patients expressed less perforin than those in healthy donors, and the percentage of perforin expressing NK cells correlated with the proportion of NKp30, NKp46, NKG2D, and DNAM-1 expressing NK cells in GC patients. Thus, it is tempting to speculate that NK cell function is systemic decreased in GC patients, which may further contribute to GC metastasis. Studies have shown that phenotypic alterations in peripheral blood NK cells are linked to tumor progression [12, 19]. In particular, low levels of NKp46 expression on circulating NK cells were associated with poor survival in stage I to III colorectal cancer patients and stage IV melanoma patients [20, 21]. Here, we also found decreased proportions of NKp46 as well as NKp30, NKG2D, and DNAM-1 expressing NK cells in stage III-IV patients compared to stage I-II patients. Unfortunately, we did not elucidate the relationship between patients' prognosis and NK cell activating receptor expression levels due to the limited cohort of GC patients examined. Hence, further studies which specifically investigate these prognostic implications with a larger patient cohort are warranted.

NK cell receptor expression varies with age and cytomegalovirus (CMV) infection status [22]. NK cells from elderly individuals are characterized by the downregulation of NKG2A and upregulation of KIRs [23]. In healthy donors with prior exposure to CMV, a subset of Fc*ε*RI-*γ*-deficient NK cells with a predominant expression of KIRs or NKG2C but decreased expression of NKp46, NKp30, and NKG2A has been reported [24–26]. However, we did not observe any phenotypic NK cell differences in terms of NKG2A and KIRs (CD158a/h, CD158b, and CD158e1) expression between GC patients and healthy donors. In addition, we found a significant decrease of NKG2D and DNAM-1 expression on NK cells in GC patients compared to healthy donors, whereas there was no significant difference in NKG2D and DNAM-1 expression between Fc*ε*RI-*γ*-deficient NK cells and conventional NK cells [26]. Healthy donors enrolled in our study were age-matched with GC patients. As the influence of CMV infection status on NK cell phenotype was closely associated with age [22], we postulated that CMV infection would not be a significant variable in this study. Nevertheless, the relationship between CMV infection status and NK cell receptor expression in GC patients merits further investigation.

TGF-*β*1 has been demonstrated to significantly decrease NK cell receptor expression and hence alter NK cell function [27–29]. Thus, we measured the concentration of plasma TGF-*β*1 in GC patients and found that it was increased in GC patients compared to healthy donors. Further analysis showed that TGF-*β*1 concentrations negatively correlated with the proportion of NKp30, NKp46, NKG2D, and DNAM-1 expressing NK cells in the peripheral blood. Subsequently, we confirmed that *in vitro* administration of TGF-*β*1 selectively downregulated NKp30, NKp46, NKG2D, and DNAM-1 expression without altering the expression of other NK cell receptors. However, a previous study reported that TGF-*β*1 could not influence the expression of NKp46 on NK cells [28]. The discrepancy of this phenomenon might be owing to the difference between that and our system because they examined the NKp46 expression after NK cells were incubated for 7 days *in vitro*. Recently, one study showed that TGF-*β*1 was able to downregulate the expression of NKp46 on NK cells [29], which was supporting our data. Altogether, these observations demonstrated that TGF-*β*1 could exert immunosuppressive effects on NK cells in GC patients. TGF-*β*1 first binds to TGF-*β* receptor II (TGF*β*R2), which induces its autophosphorylation and the phosphorylation of TGF*β*R1, then triggering the activation of SMAD2/3-dependent canonical signaling and the downstream modulation of gene expression [30]. Our data showed that galunisertib, a small-molecule inhibitor of TGF-*β*R1, was able to reverse the TGF-*β*1-induced downregulation of NKp30, NKp46, NKG2D, and DNAM-1 receptor expression on NK cells. Given that our previous studies have shown that TGF-*β*1/TGF*β*R axis is involved in immunosuppression by GC [3, 31], and that galunisertib is undergoing clinical trials in human cancers [32–34], it is possible that galunisertib may be a promising immunotherapeutic agent for the attenuation of TGF-*β*1-dependent immunosuppression in human GC.

## 5. Conclusions

In conclusion, our study has demonstrated that decreased proportions of NKp30, NKp46, NKG2D, and DNAM-1 expressing peripheral blood NK cells correlated with tumor progression in GC patients. The downregulation of NKp30, NKp46, NKG2D, and DNAM-1 receptor expression could be induced by TGF-*β*1 *in vitro*, and this effect was reversed by the addition of the TGF-*β*R1 inhibitor galunisertib. Thus, our studies suggest that galunisertib may be a useful therapeutic agent for the targeting of TGF-*β*1-induced NK cell impairment during GC.

## Figures and Tables

**Table 1:** The comparison of surface receptors on NK cells in 30 healthy donors and 30 GC patients.

Surface receptors	Healthy donors (%)	GC patients (%)	*P*
CD56^high^	2.70 ± 0.46	3.53 ± 0.44	0.195
CD16	90.97 ± 0.91	88.89 ± 1.09	0.149
NKp30	53.83 ± 4.26	35.71 ± 4.14	**0.003**
NKp46	36.45 ± 2.85	28.88 ± 2.45	**0.048**
NKG2D	96.21 ± 0.63	90.69 ± 1.56	**0.002**
DNAM-1	95.02 ± 0.61	90.57 ± 1.32	**0.021**
CD94	55.64 ± 3.52	57.94 ± 2.96	0.618
NKG2A	10.57 ± 2.49	13.27 ± 2.30	0.429
2B4	98.23 ± 0.68	97.93 ± 0.39	0.703
CD158a/h	21.98 ± 2.99	19.64 ± 2.55	0.553
CD158b	54.72 ± 2.94	47.83 ± 4.28	0.101
CD158e1	17.01 ± 2.42	18.73 ± 2.41	0.615

## Data Availability

The data used to support the findings of this study are available from the corresponding author upon request.

## Supplementary Materials

Supplementary Materials The prevalence of circulating NK cells in GC patients. (A) A representative flow cytometry analysis of CD3^−^CD56^+^ NK cells, CD3^+^CD56^+^ NKT cells, and CD3^+^CD56^−^ T cells in the peripheral blood of GC patients (GC blood) and healthy donors (normal blood). Whole peripheral blood with lysed red cells were stained with PE-Cy7 anti-human CD56 and APC anti-human CD3 antibodies, and CD3^−^CD56^+^ NK-cell, CD3^+^CD56^+^ NKT-cell, and CD3^+^CD56^−^ T-cell percentages were analyzed from the lymphocyte gate as defined by FSC and SSC dot-plot. (B) Statistical analysis of CD3^−^CD56^+^ NK-cell, CD3^+^CD56^+^ NKT-cell, and CD3^+^CD56^−^ T-cell levels in the peripheral blood of 30 GC patients and 30 healthy donors. Data were expressed as the mean ± SEM. Each plot represents a single donor. Supplementary Figure 2. CD107a degranulation of circulating NK cells from 2 GC patients and 2 healthy donors (controls). 2 × 10^5^ NK cells were cultured with K562 cells at 10 : 1 effector/target ratio for 1 h at 37°C and incubated with anti-CD107a-FITC (H4A3, BioLegend), followed by an additional 4-hour incubation in the presence of protein transport inhibitor (GolgiStop, BD Bioscience). After that, cells were washed, stained with anti-CD56-APC (MEM-188, BioLegend), and subjected to flow cytometric analysis. Supplementary Figure 3. The expression of perforin and granzyme B in circulating CD3^−^CD56^+^ NK cells of GC patients. (A) Statistical analysis of perforin^+^ and granzyme B^+^ NK-cell levels in the peripheral blood of 30 GC patients and 30 healthy donors. (B) Correlation of the percentages of perforin^+^ NK cells with the percentages of NKp30^+^, NKp46^+^, NKG2D^+^, and DNAM-1^+^ NK cells in GC patients. ^∗∗∗^, *P* < 0.001. Supplementary Figure 4. The plasma concentrations of TGF-*β*1 in 30 healthy donors (normal controls) and 30 GC patients. *P* < 0.05 was considered to be significant. Supplementary Figure 5. No alteration of CD16, CD158a/h, CD94, CD158b, NKG2A, CD158e1, and 2B4 expression on NK cells after TGF-*β*1 stimulation. Purified NK cells from healthy donors were seeded in 96-well plates supplemented with 1000 U/ml rhIL-2 for 48 hours with or without 10 ng/ml rhTGF-*β*, and the expression of CD16, CD158a/h, CD94, CD158b, NKG2A, CD158e1, and 2B4 on NK cells were detected by flow cytometry (*n* = 4). Supplementary Figure 6. The comparison of TGF-*β*1 levels between stage I-II and stage III-IV of GC patients. *P* < 0.05 was considered to be significant.
