# Supplementary material for: Altered NKp30, NKp46, NKG2D, and DNAM-1 Expression on Circulating NK Cells Is Associated with Tumor Progression in Human Gastric Cancer
Source: J Immunol Res. 2018 Sep 3;2018:6248590. doi: 10.1155/2018/6248590 (PMC6140275; doi:10.1155/2018/6248590)
Supplement: Supplementary Materials — The prevalence of circulating NK cells in GC patients. (A) A representative flow cytometry analysis of CD3−CD56+ NK cells, CD3+CD56+ NKT cells, and CD3+CD56− T cells in the peripheral blood of GC patients (GC blood) and healthy donors (normal blood). Whole peripheral blood with lysed red cells were stained with PE-Cy7 anti-human CD56 and APC anti-human CD3 antibodies, and CD3−CD56+ NK-cell, CD3+CD56+ NKT-cell, and CD3+CD56− T-cell percentages were analyzed from the lymphocyte gate as defined by FSC and SSC dot-plot. (B) Statistical analysis of CD3−CD56+ NK-cell, CD3+CD56+ NKT-cell, and CD3+CD56− T-cell levels in the peripheral blood of 30 GC patients and 30 healthy donors. Data were expressed as the mean ± SEM. Each plot represents a single donor. Supplementary Figure 2. CD107a degranulation of circulating NK cells from 2 GC patients and 2 healthy donors (controls). 2 × 10^5^ NK cells were cultured with K562 cells at 10 : 1 effector/target ratio for 1 h at 37°C and incubated with anti-CD107a-FITC (H4A3, BioLegend), followed by an additional 4-hour incubation in the presence of protein transport inhibitor (GolgiStop, BD Bioscience). After that, cells were washed, stained with anti-CD56-APC (MEM-188, BioLegend), and subjected to flow cytometric analysis. Supplementary Figure 3. The expression of perforin and granzyme B in circulating CD3−CD56+ NK cells of GC patients. (A) Statistical analysis of perforin+ and granzyme B+ NK-cell levels in the peripheral blood of 30 GC patients and 30 healthy donors. (B) Correlation of the percentages of perforin+ NK cells with the percentages of NKp30+, NKp46+, NKG2D+, and DNAM-1+ NK cells in GC patients. ∗∗∗, P < 0.001. Supplementary Figure 4. The plasma concentrations of TGF-β1 in 30 healthy donors (normal controls) and 30 GC patients. P < 0.05 was considered to be significant. Supplementary Figure 5. No alteration of CD16, CD158a/h, CD94, CD158b, NKG2A, CD158e1, and 2B4 expression on NK cells after TGF-β1 stimulation. Purified NK cel [file 6248590.f1.pdf]

Supplementary Figure 1 through 6 and Supplementary table 1

Figure S1. The prevalence of circulating NK cells in GC patients. **Figure S2.** CD107a degranulation of circulating NK cells from GC patients and healthy donors. **Figure S3.** The correlation between the percentages of Perforin^{+} NK cells and the percentages of NKp30^{+}, NKp46^{+}, NKG2D^{+} as well as DNAM-1^{+} NK cells in GC patients. **Figure S4.** The plasma concentrations of TGF-β1 in 30 healthy donors and 30 GC patients. **Figure S5.** No alteration of CD16, CD158a/h, CD94, CD158b, NKG2A, CD158e1 and 2B4 expression on NK cells after TGF-β1 stimulation. **Figure S6.** The comparison of TGF-β1 levels between stage I-II and stage III-IV of GC patients. **Table S1.** Clinical characteristics of 30 GC patients and 30 healthy donors.

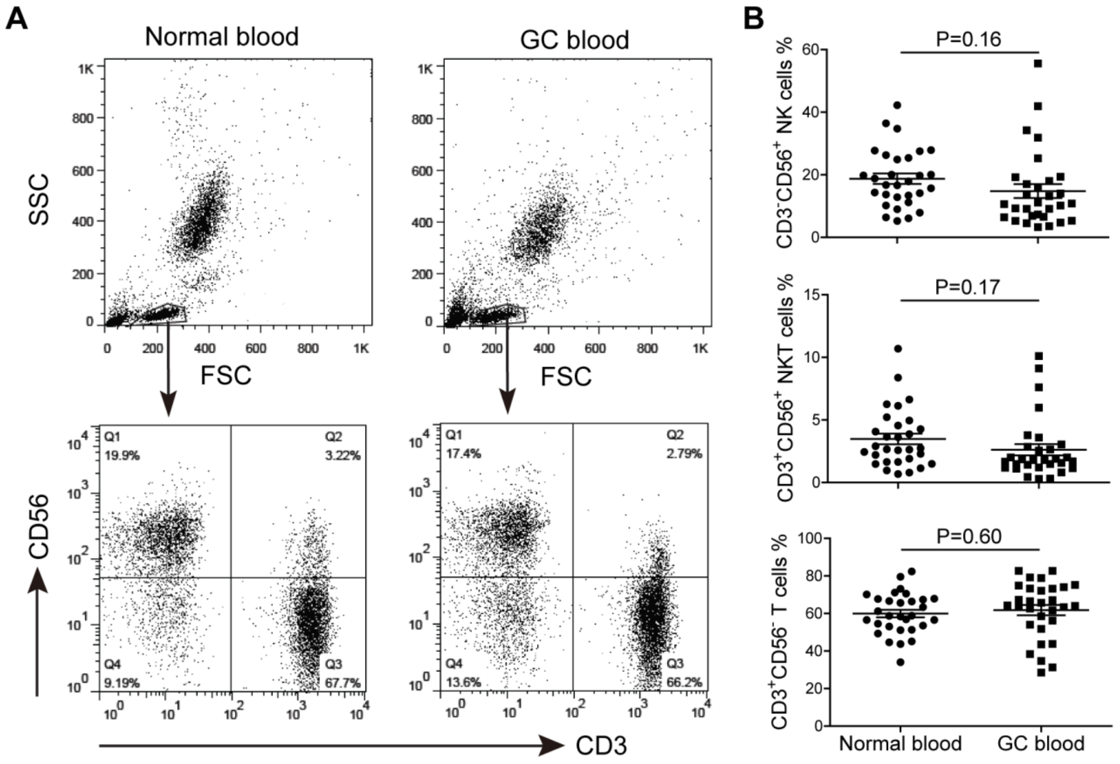

Supplementary Figure 1. The prevalence of circulating NK cells in GC patients. (A) A representative flow cytometry analysis of $CD3^-CD56^+$ NK cells, $CD3^+CD56^+$ NKT cells and $CD3^+CD56^-$ T cells in the peripheral blood of GC patients (GC blood) and healthy donors (normal blood). Whole peripheral blood with lysed red cells were stained with PE-Cy7 anti-human CD56 and APC anti-human CD3 antibodies, and $CD3^-CD56^+$ NK-cell, $CD3^+CD56^+$ NKT-cell and $CD3^+CD56^-$ T-cell percentages were analyzed from the lymphocyte gate as defined by an FSC and SSC dot-plot. (B) Statistical analysis of $CD3^-CD56^+$ NK-cell, $CD3^+CD56^+$ NKT-cell and $CD3^+CD56^-$ T-cell levels in the peripheral blood of 30 GC patients and 30 healthy donors. Data were expressed as the mean ± SEM. Each plot represents a single donor.

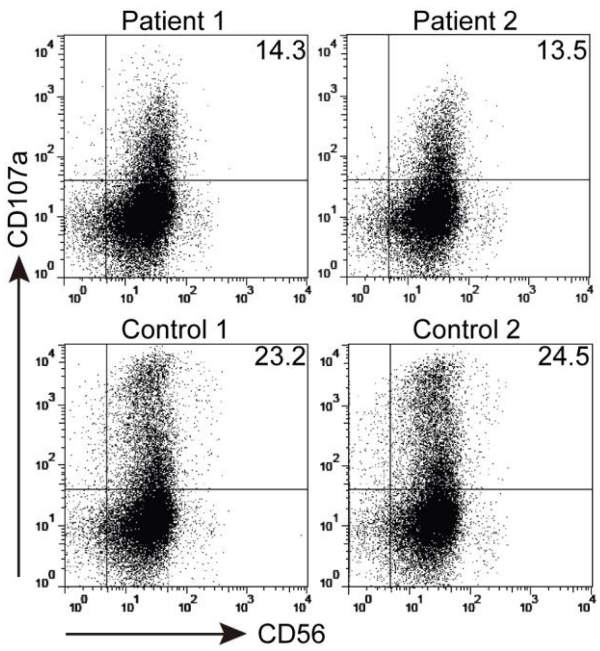

Supplementary Figure 2. CD107a degranulation of circulating NK cells from 2 GC patients and 2 healthy donors (controls). 2×10^5 NK cells were cultured with K562 cells at 10:1 effector/target ratio for 1 h at 37 ℃ and incubated with anti-CD107a-FITC (H4A3, Biolegend), followed by an additional 4-hour incubation in the presence of protein transport inhibitor (GolgiStop, BD Bioscience). After that, cells were washed, stained with anti-CD56-APC (MEM-188, Biolegend), and subjected to flow cytometric analysis.

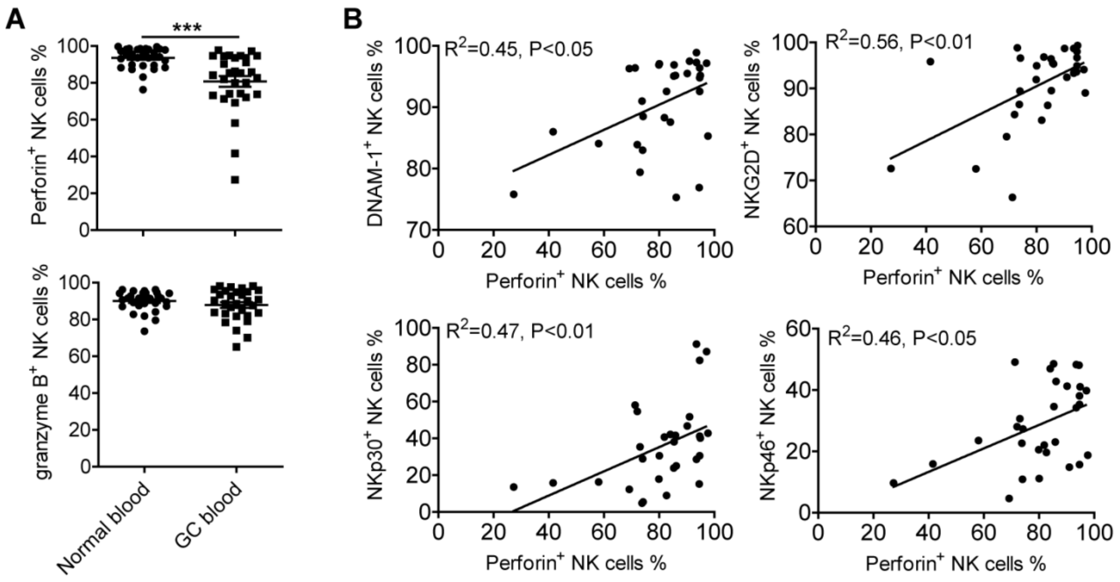

Supplementary Figure 3. The expression of Perforin and granzyme B in circulating CD3$^-$CD56$^+$ NK cells of GC patients. (A) Statistical analysis of Perforin$^+$ and granzyme B$^+$ NK-cell levels in the peripheral blood of 30 GC patients and 30 healthy donors. (B) Correlation of the percentages of Perforin$^+$ NK cells with the percentages of NKp30$^+$, NKp46$^+$, NKG2D$^+$ and DNAM-1$^+$ NK cells in GC patients. ***, P<0.001.

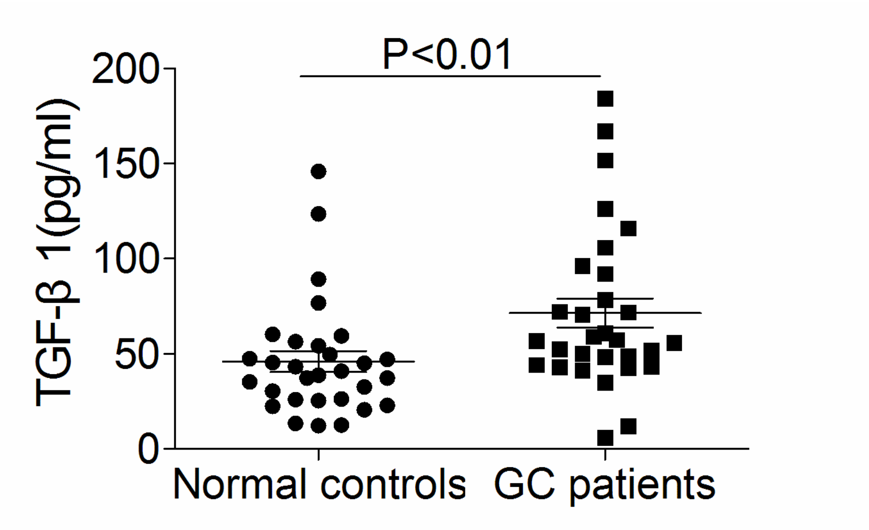

Supplementary Figure 4. The plasma concentrations of TGF-β1 in 30 healthy donors (normal controls) and 30 GC patients. *P*<0.05 was considered to be significant.

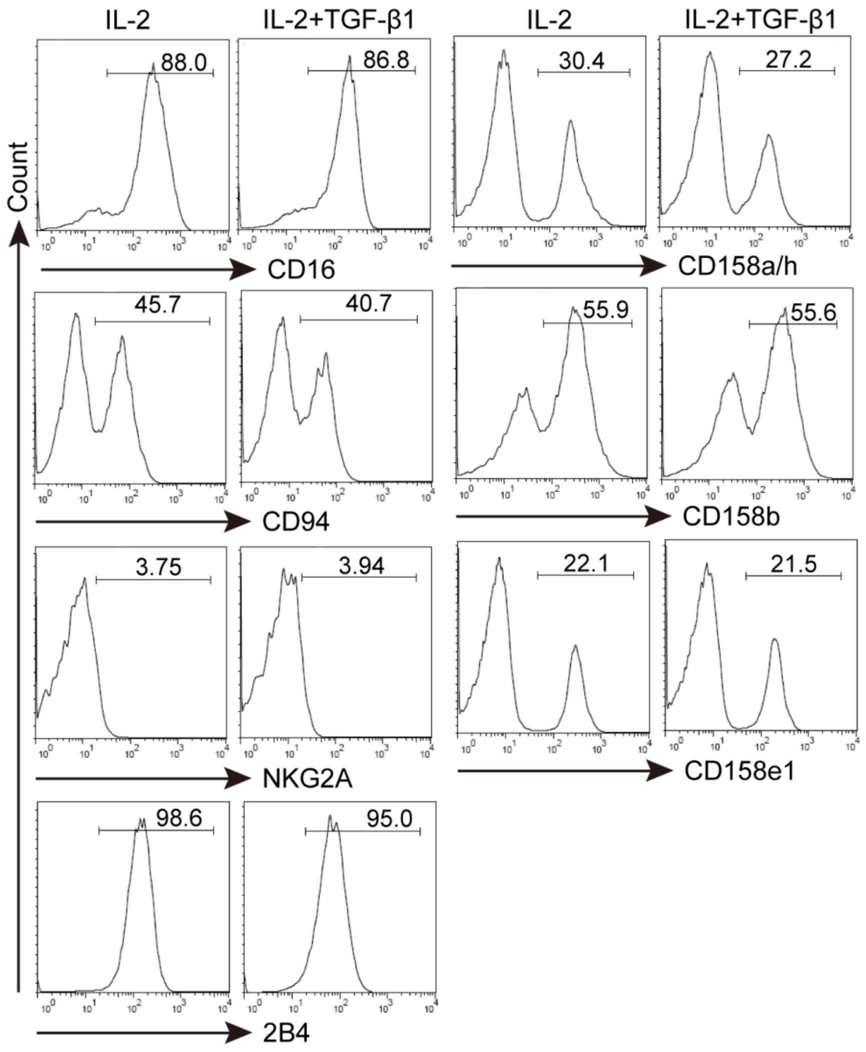

Supplementary Figure 5. No alteration of CD16, CD158a/h, CD94, CD158b, NKG2A , CD158e1 and 2B4 expression on NK cells after TGF-β1 stimulation. Purified NK cells from healthy donors were seeded in 96-well plates supplemented with 1000 U/ml rhIL-2 for 48 hours with or without 10 ng/ml rhTGF-β, and the expression of CD16, CD158a/h, CD94, CD158b, NKG2A , CD158e1 and 2B4 on NK cells were detected by flow cytometry (n=4).

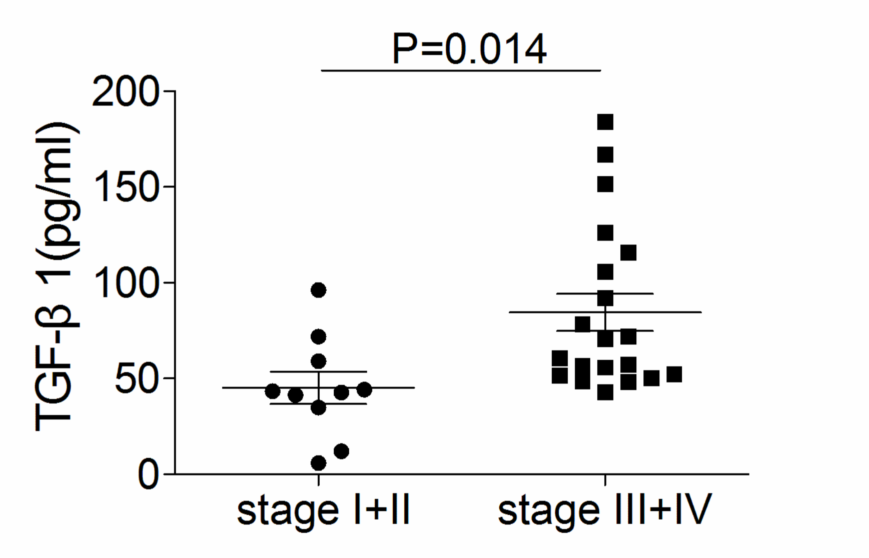

Supplementary Figure 6. The comparison of TGF-β1 levels between stage I-II and stage III-IV of GC patients. $P<0.05$ was considered to be significant.

Supplementary Table 1 Clinical characteristics of 30 GC patients and 30 healthy donors

Variables	GC patients	healthy donors
Sex (male/female)	20/10	20/10
Age (*y*), median (range)	51, 28-75	51, 28-75
Tumor (T) invasion (T1/T2/T3/T4)	2/5/2/21	0
Lymphoid nodal (N) status (N0/N1/N2/N3)	7/2/7/14	0
Distant metastasis (M) status (M0/M1)	28/2	0
TNM stage (Ⅰ/Ⅱ/III/Ⅳ)	3/7/18/2	0
Histologic type (good/moderate/poor)	0/10/20	0
